# Putting into Practice

**Published:** 1892-07-23

**Authors:** 


### Passing Topics.

### PUTTING INTO PRACTICE.

The announcement that it is Lord Sandhurst's intention, so soon as the turmoil of the General Election is over, to take steps towards the constitution of the proposed Central Council is both interesting and welcome. Obviously, if anything practical is to come of the labour of the Lords' Select Committee, it is needful that their recommendations should be dealt with promptly in order that advantage may be taken of the existence of such interest in hospital affairs, all too little we fear, as the publication of the Report has generated. It is a lamentable circumstance that so far the only result of the enquiry appears to have been a disposition to make it serve as an excuse for withholding assistance from the institutions, and this notwithstanding its favourable issue. Many well-meaning people are only too ready to enter upon that compromise with conscience which consists of waiting upon circumstances. At present the minds of many supporters of hospitals have been disturbed. The fact that an enquiry was instituted seemed to suggest that all was not quite as it should be, and we fear that, notwithstanding the testimony the Committee has offered to the value and general efficiency of the hospitals, memory will be disposed to favour the circumstance that charges were made rather than that they were satisfactorily disposed of.

There is no question of rehabilitating hospitals in public estimation. Unhappily they have never stood very high there, except perhaps in a purely sentimental sense. No doubt it has been the fashion to speak and to think well of them, but this platonic regard has not gone very far, and the history of our voluntary hospitals, so far as the bulk of the people are concerned, is a history of neglect, except upon the part of those who have made use of them. The point to be kept in view, even if this requires constant reiteration, is the necessity for bringing home our hospital work to the consciences of the multitude. The present state of the Hospital Sunday Fund affords a fitting illustration. The total sum collected to this time, notwithstanding the strenuous efforts made and the help afforded by the publication of Mr. Egmont Hake's "Suffering London," falls considerably short of the total of last year. The aggregate of the congregational offertories may be a little more, but as regards the late Duke of Cleveland's gift in 1891, the fund is wholly deficient. That is to say, the collection last year depended upon a single individual among a population of five millions for one-eighth part of the amount realised, just as it depends regularly for a similar proportion upon the efforts of three or four energetic and whole-hearted clergymen out of many hundreds, most, of course, with less, but some undoubtedly with equal opportunities; and because an individual similarly disposed and capable cannot be found this year, the fund is the poorer by £5,000.

We are aware it is not permitted to us to say too much, or it may be objected that we are presuming to scold people for not performing that which if performed at all is a purely voluntary act. But as the Lords have unhesitatingly pronounced in favour of the present system of medical relief, we can do no harm by endeavouring to enforce, though at the risk of repetition, the necessity for discovering the right way of making the population as a whole sensitive to their hospital duties and privileges. It is in this direction of all others that it seems to us the Central Council may be made useful. Agricola.

